# Society Meetings

**Published:** 1905-05

**Authors:** 


### SOCIETY MEETINGS.

THE Buffalo Academy of Medicine held meetings during the month of April as follows:

*Section on Surgery.*—Tuesday evening, April 4. Program: (*a*) Congenital dislocations of the hip, Bernard Bartow; (*b*) orificial surgery: its absurdities, philosophy and merit, Herman Hayd.

*Section on Medicine.*—Tuesday evening, April 11. Program: (*a*) The future viewpoint and practice of infant feeding, Henry Dwight Chapin, professor of diseases of children in the Post-Graduate Medical School, New York City; discussion opened by Irving M. Snow; (*b*) the treatment of disease by means other than drugs, A. W. Bayliss. General discussion.

*Section on Pathology.*—Tuesday evening, April 18.—Program: Mechanical factors of urine formation, Torald Sollmann, Cleveland, O.

*Section on Obstetrics.*—Tuesday evening, April 25. Program: Cesarean section, Charles E. Congdon.

THE Esculapian Club held its regular monthly meeting at the Hotel Touraine, Thursday evening, April 20, 1905, at 8.30. The host was Dr. J. B. Young. The paper of the evening was the presidential address of Dr. C. E. Congdon.

THE American Academy of Ophthalmology and Oto-Laryngology will hold its next annual meeting in Buffalo, September 14, 15 and 16, 1905. The committee of arrangements is: Alvin

A. Hubbell, chairman; F. Park Lewis, Benjamin H. Grove, W. Scott Renner, of Buffalo, and Sherman Voorhees, of Elmira. The date has been changed from August 23-25, to that above mentioned.

---

THE Buffalo Ophthalmological Club will hold its next meeting at Ithaca, May 3, 1905, as the guests of the nonresident members, Dr. Kirkendall, of Ithaca, and Drs. Case and Voorhees, of Elmira.

---

THE American Gynecological Society will hold its thirtieth annual meeting at the Cataract House, Niagara Falls, Thursday, Friday, and Saturday, May 25, 26, and 27, 1905, under the presidency of Dr. E. C. Dudley, of Chicago.

